# Role of serotonin neurons in the dorsal raphe nucleus in heroin self-administration and punishment

**DOI:** 10.1038/s41386-024-01993-1

**Published:** 2024-09-19

**Authors:** Chen Li, Nicholas S. McCloskey, Saadet Inan, Lynn G. Kirby

**Affiliations:** Center for Substance Abuse Research, Lewis Katz School of Medicine at Temple University, Philadelphia, USA

**Keywords:** Addiction, Stress and resilience

## Abstract

One hallmark of substance use disorder is continued drug use despite negative consequences. When drug-taking behavior is punished with aversive stimuli, i.e. footshock, rats can also be categorized into punishment-resistant or compulsive vs. punishment-sensitive or non-compulsive phenotypes. The serotonin (5-hydroxytryptamine, 5-HT) system modulates responses to both reward and punishment. The goal of the current study was to examine punishment phenotypes in heroin self-administration and to determine the role of dorsal raphe nucleus (DRN) 5-HT neurons in both basal and punished heroin self-administration. First, rats were exposed to punished heroin self-administration and neuronal excitability of DRN 5-HT neurons was compared between punishment-resistant and punishment-sensitive phenotypes using ex vivo electrophysiology. Second, DRN 5-HT neuronal activity was manipulated in vivo during basal and punished heroin self-administration using chemogenetic tools in a Tph2-iCre rat line. While rats separated into punishment-resistant and punishment-sensitive phenotypes for punished heroin self-administration, DRN 5-HT neuronal excitability did not differ between the phenotypes. While chemogenetic inhibition of DRN 5-HT neurons was without effect, chemogenetic activation of DRN 5-HT neurons increased both basal and punished heroin self-administration selectively in punishment-resistant animals. Additionally, the responsiveness to chemogenetic activation of DRN 5-HT neurons in basal self-administration and motivation for heroin in progressive ratio each predicted resistance to punishment. Therefore, our data support the role for the DRN 5-HT system in compulsive heroin self-administration.

## Introduction

Opioid use disorder (OUD) is a rising public health crisis characterized by significant clinical impairment and distress [1, 2]. A hallmark of opioid use disorder is compulsive drug use despite negative consequences which may result from both physical dependence and neural system dysregulation [3, 4]. Animal models with improved translational validity are needed to develop more effective treatment strategies for OUDs [5, 6].

Punished self-administration procedures have been developed to model human compulsive drug use or drug-taking that persists despite negative consequences [7–9]. Like human drug users, only a subset of animals continues to self-administer drugs when contingent footshock is paired with drug delivery [punishment-resistant, compulsive], while others significantly decrease or stop drug intake [punishment-sensitive, non-compulsive] [7, 8]. These divergent behavioral patterns in punishment procedures have been observed in response to multiple drugs of abuse including alcohol, cocaine, methamphetamine, and oxycodone [10–16]. In this study, we further expand the punishment model to heroin self-administration. Additionally, our study examines the role of dorsal raphe nucleus (DRN) serotonin (5-hydroxytryptamine, 5-HT) neurons in basal and punished heroin self-administration.

The midbrain 5-HT DRN contains the largest population of 5-HT neurons that target many functionally distinct brain regions [17]. Evidence shows that the DRN regulates responses to both rewarding and aversive stimuli [18, 19]. For example, in vivo fiber photometry studies indicate that DRN 5-HT neurons respond to the anticipatory and consummatory phases of reward while DRN 5-HT and GABA neurons respond to aversive stimuli [20, 21]. Optogenetic manipulation of DRN neuronal activity produces reward-related behaviors [22, 23], modifies motivation to acquire rewards [24], and affects behavioral flexibility in a win-stay (reward-sensitive) and lose-shift (punishment-sensitive) probabilistic reversal learning task [25, 26]. Our previous studies in rats showed that the DRN is necessary for both involuntary footshock-induced 22 kHz ultrasonic vocalizations (distress calls) and for swim stress-induced opioid reinstatement in a morphine-conditioned place-preference model [27, 28]. Moreover, our laboratory has recently found that exposure of rats to early life stress produces enduring suppression of DRN 5-HT neuronal excitability and also elevates alcohol intake in adulthood (under revision). Therefore, we hypothesized that the DRN 5-HT neurons may regulate heroin taking behavior in a punishment model that presents a conflict of lever pressing with both rewarding and aversive consequences.

To test our hypothesis, we first established a heroin punishment model in rats and compared DRN 5-HT neuronal excitability between two phenotypes (punishment-resistant vs punishment-sensitive) via ex vivo electrophysiology. Second, we used designer receptors exclusively activated by designer drugs (DREADDs) to manipulate DRN 5-HT neuronal activity specifically in a rat line expressing Cre under a tryptophan hydroxylase 2 (Tph2) promoter and investigated a role for the DRN 5-HT system in basal and punished heroin self-administration.

## Methods

### Subjects

#### Experiment 1

Male Sprague-Dawley rats (Taconic Farms, Germantown, NY) arrived in the laboratory at the age of 7–8 weeks and were housed 2 per cage under standard temperature (20°C) and humidity (40%) on a reversed 12 h light/dark cycle (lights off at 8:00 AM), with food and water provided *ad libitum*. After 6–7 days of acclimation, rats received i.v. catheter surgeries [29], and were singly housed throughout the rest of the experiment.

#### Experiment 2

Tph2-iCre rat breeders on Sprague-Dawley background were generously provided by Drs. Katinka Stecina and Larry Jordan at the University of Manitoba, Canada, and originally developed by Drs. Dusan Bartsch and Kai Schoenig [30]. Upon weaning, same-sex offspring were housed 2–4 per cage with enrichment until the age of 8–9 weeks. Hemizygous Tph2-iCre males received intra-DRN virus injection and i.v. catheter implantation and were singly housed throughout the rest of the experiment.

Animal protocols were approved by the Temple University Institutional Animal Care and Use Committee and were conducted in accordance with the National Research Council *Guide for the Care and Use of Laboratory Animals*.

### Intravenous catheter implantation

Rats were anesthetized, a polyurethane i.v. catheter was implanted into the right jugular vein, and patency was confirmed and maintained as previously described [29, 31]. The analgesic ketoprofen (5 mg/kg) was given subcutaneously prior to surgery and on postoperative day 1. Two weeks of recovery were given for experiment 1 before self-administration.

### Intracranial virus injection

On the same day of i.v catheter implantation, rats in experiment 2 also received an intra-DRN (AP: −7.6, ML: 3.8, DV: −7.4, at 26-degree angle) injection of 2 ul adeno-associated virus (AAV) vectors [AAV8-hSyn-DIO-hM3D(Gq)-mCherry (≥ 4 × 10^12^ vg/mL), AAV8-hSyn-DIO-hM4D(Gi)-mCherry (≥ 1 × 10^13^ vg/mL) or AAV8-hSyn-DIO-mCherry (≥ 1 × 10^13^ vg/mL); Addgene, Watertown, MA] via a 33-gauge injector at a speed of 0.2 μl/min. Two weeks were allowed for virus expression before tamoxifen (40 mg/kg, i.p.) (Sigma-Aldrich, St. Louis, MO) was injected daily for 5 days to activate iCre. Self-administration started 1 week after tamoxifen treatment. Viral injections were confirmed by immunohistochemistry for each rat at the end of the experiment. Behavioral data were included only in those rats with robust mCherry expression confined to the DRN (see Fig. 2B and Supplementary Fig S5).

### Experimental design

#### Experiment 1

**Fig. 1:**
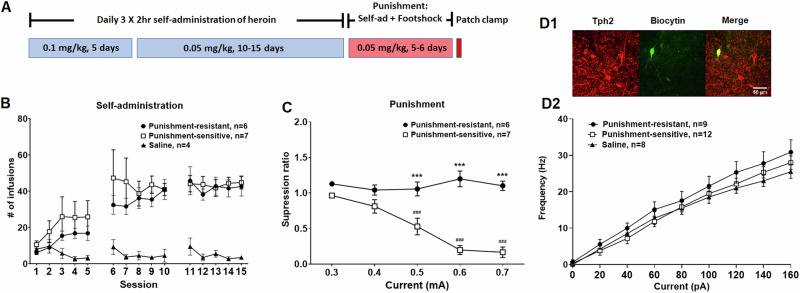
Punishment separated rats into punishment-resistant and punishment-sensitive phenotypes, which were not associated with excitability of DRN 5-HT neurons. **A** Experimental timeline of heroin self-administration training and punishment. Rats were trained to self-administer heroin with daily 3 × 2 h sessions at an initial dose of 0.1 mg/kg/infusion for 5 days and maintained at 0.05 mg/kg for 10 days. During the punishment, footshocks were paired with all drug deliveries, and the intensity of footshocks was increased daily. Excitability of the DRN 5-HT neurons was recorded the next day after the last punishment session. **B** punishment-resistant and punishment-sensitive rats took similar amounts of heroin during the self-administration acquisition phase. **C** An ascending intensity of footshocks decreased heroin self-administration in punishment-sensitive rats but not punishment-resistant rats. Pound signs indicate that heroin intake in punishment-sensitive rats significantly dropped from punishment day 1 (### *p* ≤ 0.001, Tukey test). Asterisks indicate a significant difference between punishment-sensitive and punishment-resistant rats on punishment days 3–5 (***p ≤ 0.001, Tukey test). **D** Excitability of DRN 5-HT neurons did not differ between punishment-resistant and punishment-sensitive phenotypes and was unaffected by heroin history. (D1) *Post hoc* immunohistochemical identification of the biocytin-filled recorded neuron (green) with Tph2-IR (red). (D2) Excitability curves (current-induced action potential frequency) were similar in punishment-resistant and punishment-sensitive subgroups and a saline control group not exposed to punishment.

The behavioral timeline for experiment 1 is shown in Fig. 1A. Self-administration procedures are detailed in Supplementary methods as described previously [31]. Rats were trained with a daily 3 × 2 h long access session, and each 2 h session was separated by a 30 min interval during which rats remained in self-administration chambers with both levers retracted and house lights turned off [15]. Rats were randomly assigned to heroin or saline groups. For heroin rats, active lever presses were reinforced with a fixed ratio (FR) 1 of heroin infusion. For saline rats, active lever presses resulted in saline infusion. Rats self-administered heroin at a dose of 0.1 mg/kg/infusion for 5 sessions. Then the dose was halved to 0.05 mg/kg/inf for another 10 self-administration sessions before the punishment phase began. The next day after the last self-administration session, saline rats were tested for DRN 5-HT neuronal excitability with electrophysiology and heroin rats continued to the punishment phase.

For punishment sessions, active lever presses triggered identical consequences as basal self-administration with the addition of a 0.5 s footshock delivered through the grid floor. The footshock came immediately after the lever press and 100% of drug infusions were punished. A series of ascending shock intensity was applied, with the initial intensity at 0.3 mA, and increased by 0.1 mA each day to the final intensity at 0.7 mA over the course of 5 days. The next day after the last punishment session, heroin rats were euthanized and brains were prepared for assessment of DRN 5-HT neuronal excitability with electrophysiology.

#### Experiment 2

The behavioral timeline for experiment 2 is shown in Fig. 3A, Tph2-iCre Rats went through FR, progressive ratio (PR), and punishment phases. After the rats established stable FR self-administration on week three (see Fig. 1B and 3B1), on day 14, an i.p. injection of saline was given 30 min before each 2 h session. The next day, 2 mg/kg CNO (i.p.) was injected 30 min before each 2 h session. Two days after CNO treatment, a subset of 8 rats were tested with a lower dose of 0.025 mg/kg/infusion heroin in FR.

PR required higher numbers of active lever presses to receive the next infusion of heroin (see Supplementary Methods). The session was aborted if the rats did not acquire any infusion within 30 min or if the 6 h maximum session duration was reached [32]. When a stable pattern was reached (±2 infusions for two days), the rats were tested with saline pretreatment followed by CNO pretreatment the next day.

After PR, rats were placed back to the FR schedule for 1 day, and the punishment phase started the following day. Since we observed that Tph2-iCre rats were more reactive to the footshock than vendor-purchased Sprague-Dawley rats in Experiment 1, the punishment protocol was optimized for these rats. Half of the heroin infusions (50%) were randomly paired with footshock with the initial intensity as 0.3 mA and 0.4 mA for the rest of the punishment phase. On punishment day 3, rats were tested with saline followed by CNO the next day. Two days after the CNO test, a lower dose of 0.025 mg/kg of heroin was tested in punishment. At the end of the experiment, the paw withdrawal threshold was measured in a subset of rats (punishment-resistant: *n* = 5; punishment-sensitive: *n* = 4) with the Von Frey test as previously described [33] (see Supplementary Methods) to compare the pain sensitivity between punishment phenotypes.

### Electrophysiology

Electrophysiology was conducted as described previously [31] (see Supplementary Methods). Cell excitability was assessed by recording voltage responses to a series of current pulses (−100 pA to 160 pA, step +20 pA, duration 500 ms). To verify DREADD function, following establishment of a stable 10 m baseline, effects of 10 µM bath-applied CNO on membrane potential were recorded in mCherry+ DRN neurons. Only electrophysiological data from 5-HT cells confirmed by *post hoc* immunohistochemistry (See Supplementary Methods) were included in the analysis.

### Data analysis

For the experiment 1, suppression ratio during punishment sessions was defined as a number of infusions on punishment day/average number of infusions over the last three days of self-administration [16]. For the experiment 2, suppression ratio was defined as number of infusions on punishment day/self-administration baseline before punishment. Suppression ratio data from punishment day 5 in the experiment 1 and from punishment day 3 in the experiment 2 were used as inputs for TwoStep Cluster Analysis in SPSS (IBM Corporation, Armonk, NY) to classify the rats into punishment-resistant vs. punishment-sensitive phenotype. Validity of the clustering solution was assessed by silhouette measure of cohesion and separation. All other data were analyzed using GraphPad Prism (Dotmatics, San Diego, CA). Behavioral and electrophysiological data were analyzed using one or two-way repeated measures analysis of variance (ANOVA). Tukey’s multiple comparisons and Fisher’s PLSD tests were used for *post hoc* analyses when appropriate. The correlation was analyzed using simple linear regression.

## Results

### Experiment 1

#### Punishment-resistant and punishment-sensitive phenotypes in footshock-punished heroin self-administration

Figure 1 shows the timeline and results from basal and footshock-punished heroin self-administration. Figure 1B shows that all rats acquired stable self-administration of heroin but not saline. Using suppression ratio data from day 5 of the punishment phase, unbiased TwoStep Cluster Analysis (average silhouette score = 0.8, Fig S1A) revealed two populations of punishment responses (infusion numbers shown in Fig S2A): rats with a punishment-resistant phenotype (46%, *n* = 6) continued to take a similar amount of heroin during the punishment phase compared to baseline (mean suppression ratio 1.10 ± 0.07) and rats with a punishment-sensitive phenotype (54%, *n* = 7) decreased their drug intake during punishment by 83% (mean suppression ratio 0.16 ± 0.07) (1C). The two punishment phenotypes did not differ in their heroin self-administration at baseline, when no footshocks were presented (1B). A two-way repeated-measures ANOVA of heroin infusion numbers at baseline revealed the main effects of session [F(14,196) = 10.58, *p* ≤ 0.001] and group [F(2,14) = 9.115, *p* ≤ 0.01], and an interaction between session and group [F(28,196) = 3.126, *p* ≤ 0.001]. Post hoc Tukey tests showed that heroin groups differed from the saline group (punishment-resistant vs. saline, *p* ≤ 0.05; punishment-sensitive vs. saline, *p* ≤ 0.01), but there were no differences between punishment-resistant and punishment-sensitive groups during basal self-administration (*p* > 0.05).

During punished self-administration (1C), a two-way repeated-measures ANOVA of suppression ratio revealed the main effects of session [F(4,44) = 13.43, *p* ≤ 0.001] and punishment phenotype [F(1,11) = 50.49, *p* ≤ 0.001], and an interaction between session and punishment phenotype [F(4,44) = 17.88, *p* ≤ 0.001]. Post hoc Tukey tests showed that drug intake in punishment-sensitive rats on punishment days 3, 4, and 5 was significantly lower than on day 1 and were significantly lower than punishment-resistant rats (*p* ≤ 0.001). Drug intake in punishment-resistant rats did not change throughout the punishment phase (day 5 vs. day 1, *p* > 0.05).

#### Intrinsic excitability of the DRN 5-HT neurons did not correlate with drug history or punishment phenotype

The DRN 5-HT neurons in all three groups [saline (8 cells from 4 rats), punishment-resistant (9 cells from 5 rats), and punishment-sensitive (12 cells from 6 rats)] exhibited similar intrinsic excitability (1D). Two-way repeated-measures ANOVA of the action potential frequency revealed a main effect of current [F(8, 208) = 257.2, *p* ≤ 0.001], but neither an effect of group [F (2, 26) = 0.9022, *p* > 0.05] nor an interaction between current and group [F (16, 208) = 0.7989, *p* > 0.05].

### Experiment 2

#### Validating the Tph2-iCre rat line and DREADD function in 5-HT DRN neurons

**Fig. 2:**
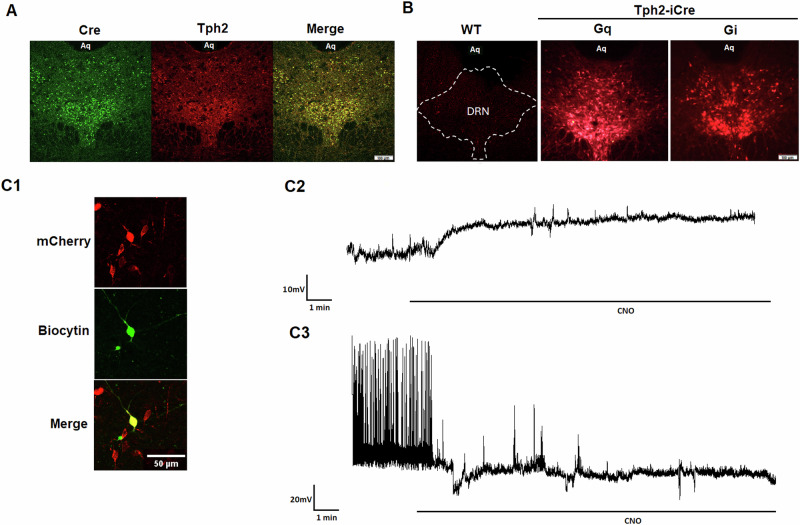
Validation of the Tph2-iCre rat strain and DREADD function in DRN 5-HT neurons. **A** Colocalization of Cre (green) and Tph2-IR neurons (red) in tamoxifen-induced Tph2-iCre rats. **B** Cre-dependent DREADDs (mCherry+) expressed in Tph2-iCre rats but not wild-type littermates. **C** DREADD function verified with electrophysiology. **C1** DREADD expression confirmed with mCherry labeling in a biocytin-filled recorded neuron (green). **C2** In vitro activation of Gq DREADDs via bath-application of CNO (10 μM) depolarized an mCherry+ DRN neuron. **C3** In vitro activation of Gi DREADDs hyperpolarized the membrane potential and inhibited neuronal activity in a mCherry+ DRN neuron.

The 5-HT specific expression of Cre recombinase in the DRN of tamoxifen-induced Tph2-iCre rats was validated by immunohistochemistry and DREADD function was verified by electrophysiology (Fig. 2). Cre expression in the DRN of Tph2-iCre rats significantly overlapped with Tph2+ neurons (2A). Following intraDRN AAV injections of Cre-dependent, mCherry-tagged DREADDs, mCherry expression was observed selectively in Tph2-iCre rats but not WT littermates (2B). Ex vivo whole-cell recordings in DRN brain slices from Tph2-iCre rats with AAV DREADD delivery showed that activating Gq or Gi DREADDs by bath application of CNO (10 μM) excited or inhibited mCherry+ (2C1) DRN neurons, respectively (2C2, 2C3).

#### Chemogenetic activation of 5-HT DRN neurons increased heroin intake in FR self-administration at a dose on the descending limb of the heroin dose-effect curve

**Fig. 3:**
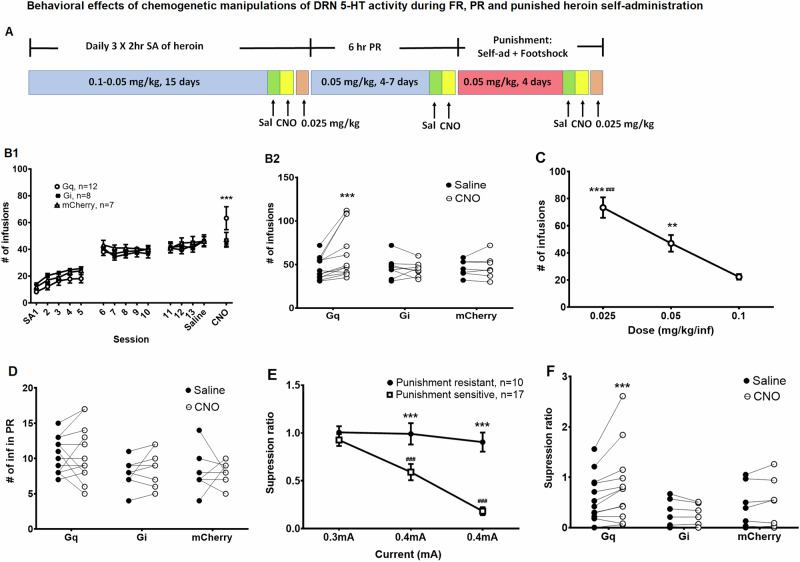
Activation of DRN 5-HT neurons in tamoxifen-induced Tph2-iCre rats increased heroin intake in FR and punished, but not PR heroin self-administration. **A** Experimental timeline of DREADD manipulations of DRN 5-HT activity during FR, PR and punished self-administration phases. **B** Activation of DRN 5-HT neurons increased heroin intake while inhibition of the DRN 5-HT neuron had no effects in FR heroin self-administration. **B1** Number of heroin infusions taken over 15 sessions of heroin self-administration. Three DREADD groups showed similar behavioral patterns of acquisition and maintenance of heroin self-administration. When treated with CNO (2 mg/kg), only the Gq group increased their number of infusions. **B2** Number of heroin infusions during FR sessions in saline- vs. CNO-treated animals in Gq, Gi, and mCherry groups. Asterisks indicate a significant difference between saline vs CNO in Gq group (***p ≤ 0.001, Tukey test). **C** Rats increased their number of infusions in FR when the dose of heroin decreased. Asterisks indicate significant differences from 0.1 mg/kg/inf (***p ≤ 0.001, **p ≤ 0.01, respectively; Tukey tests). Pound signs indicate a significant difference from 0.05 mg/kg/inf (##*p* ≤ 0.01; Tukey test). **D** Number of infusions during the PR phase in saline- and CNO-treated rats. Chemogenetic manipulation of DRN 5-HT neuronal activity did not change heroin intake in PR heroin self-administration. **E** Footshocks decreased heroin self-administration in punishment-sensitive rats but not punishment-resistant rats. Pound signs indicate that heroin intake in punishment-sensitive rats significantly dropped from punishment day 1 (###*p* ≤ 0.001; Tukey test). Asterisks indicate a significant difference between punishment-resistant and punishment-sensitive rats on punishment days 2 and 3 (***p ≤ 0.001, Tukey tests). **F** Activation of DRN 5-HT neurons increased heroin intake while inhibition of the DRN 5-HT neuron had no effects in punishment. Suppression ratio during punishment sessions in saline- vs. CNO-treated animals in Gq, Gi, and mCherry groups. Asterisks indicate a significant difference between saline vs CNO in the Gq group (***p ≤ 0.001, Tukey test).

Figure 3 shows the timeline and behavioral results of manipulating DRN 5-HT neuronal activity in FR, PR, and punished heroin self-administration. All three DREADD groups (Gq, *n* = 12; Gi, *n* = 8; mCherry controls, *n* = 7) exhibited similar self-administration of heroin (0.05 mg/kg/infusion) in the FR phase (3B1). Gq-activation of 5-HT DRN neurons increased the number of heroin infusions but Gi-inhibition had no effect (3B1, 3B2). Two-way repeated-measures ANOVA of infusion numbers revealed an interaction between session (saline vs. CNO) and DREADDs (Gq, Gi, and mCherry) [F (2, 24) = 5.380, *P* ≤ 0.05] but no other main effects [Session: F (1, 24) = 4.221, *p* > 0.05; DREADDs: F (2, 24) = 0.9351, *p* > 0.05]. Post hoc Tukey test showed a significant difference between CNO and saline treatment in the Gq group (*p* ≤ 0.001) but not in the Gi or mCherry groups (*p* > 0.05). In the absence of chemogenetic manipulations, a test of the dose-response curve for heroin self-administration in a subset of 8 rats showed that 0.05 mg/kg/inf was on the descending limb of heroin dose-response curve (3C). One-way repeated-measure ANOVA revealed a main effect of dose [F (2, 14) = 35.68, *p* ≤ 0.001], and post hoc Tukey test showed that infusion number at 0.025 mg/kg/inf was higher than at 0.05 and 0.1 mg/kg/inf (*p* ≤ 0.05 and *p* ≤ 0.001, respectively), infusion number at 0.05 mg/kg/inf was higher than at 0.1 mg/kg/inf (*p* ≤ 0.01). In PR, manipulating DRN 5-HT activity did not change number of heroin infusions (3D) or breakpoint (Fig S3A).

#### Chemogenetic activation of DRN 5-HT neurons increased heroin intake in punished self-administration

The introduction of footshock-contingent heroin self-administration separated Tph2-iCre rats into punishment-resistant and punishment-sensitive phenotypes (3E). TwoStep Cluster Analysis on suppression ratio on punishment day 3 classified the rats into 37% punishment-resistant (mean suppression ratio 0.91 ± 0.10, *n* = 10) and 63% punishment-sensitive (mean suppression ratio 0.18 ± 0.04, *n* = 17) (average silhouette score = 0.7, Fig S1B). Infusion numbers of the two phenotypes are shown in Fig S2B. Two-way repeated measures ANOVA of suppression ratio revealed the main effects of session [F (2, 50) = 40.63, *p* ≤ 0.001] and stress phenotype [F (1, 25) = 17.06, *p* ≤ 0.001], and an interaction between session and stress phenotype [F (2, 50) = 23.17, *p* ≤ 0.001]. Post hoc Tukey tests showed that heroin intake in punishment-sensitive rats on punishment days 2 and 3 was significantly lower than on day 1 and was significantly lower than punishment-resistant rats during those sessions (*p* ≤ 0.001). Heroin intake in punishment-resistant rats did not change throughout the punishment phase (day 3 vs. day 1, *p* > 0.05). Activating DRN 5-HT neurons increased drug intake during punishment while inhibiting DRN 5-HT neurons had no effects (3 F). Two-way repeated-measures ANOVA of suppression ratio revealed an interaction between session (saline vs. CNO) and DREADDs (Gq, Gi, vs. mCherry) [F (2, 24) = 4.198, *p* ≤ 0.05] but no main effects [Session: F (1, 24) = 3.933, *p* > 0.05; DREADDs: F (2, 24) = 2.608, *p* > 0.05]. Post hoc Tukey tests showed a significant difference between CNO and saline treatment in the Gq group (*p* ≤ 0.001) but not in Gi or mCherry groups (*p* > 0.05).

To consider the possibility that punishment phenotype could reflect differential sensitivity of rats to the footshock stimulus, mechanical paw sensitivity was tested in a subset of rats from experiment 2 using the Von Frey test (see Supplementary Methods). No differences in paw withdrawal threshold were found between the two phenotypes (punishment-resistant: 17.20 ± 2.47 grams, *n* = 5; punishment-sensitive: 18.00 ± 0.00 grams, *n* = 4). These data are consistent with prior studies using punishment models showing that the punishment-resistant phenotype is not driven by lower pain sensitivity [34, 35].

#### Rats with punishment-resistant and punishment-sensitive phenotypes responded differently to chemogenetic activation of DRN 5-HT neurons in FR and punishment as well as lowering the dose of heroin during punishment

**Fig. 4:**
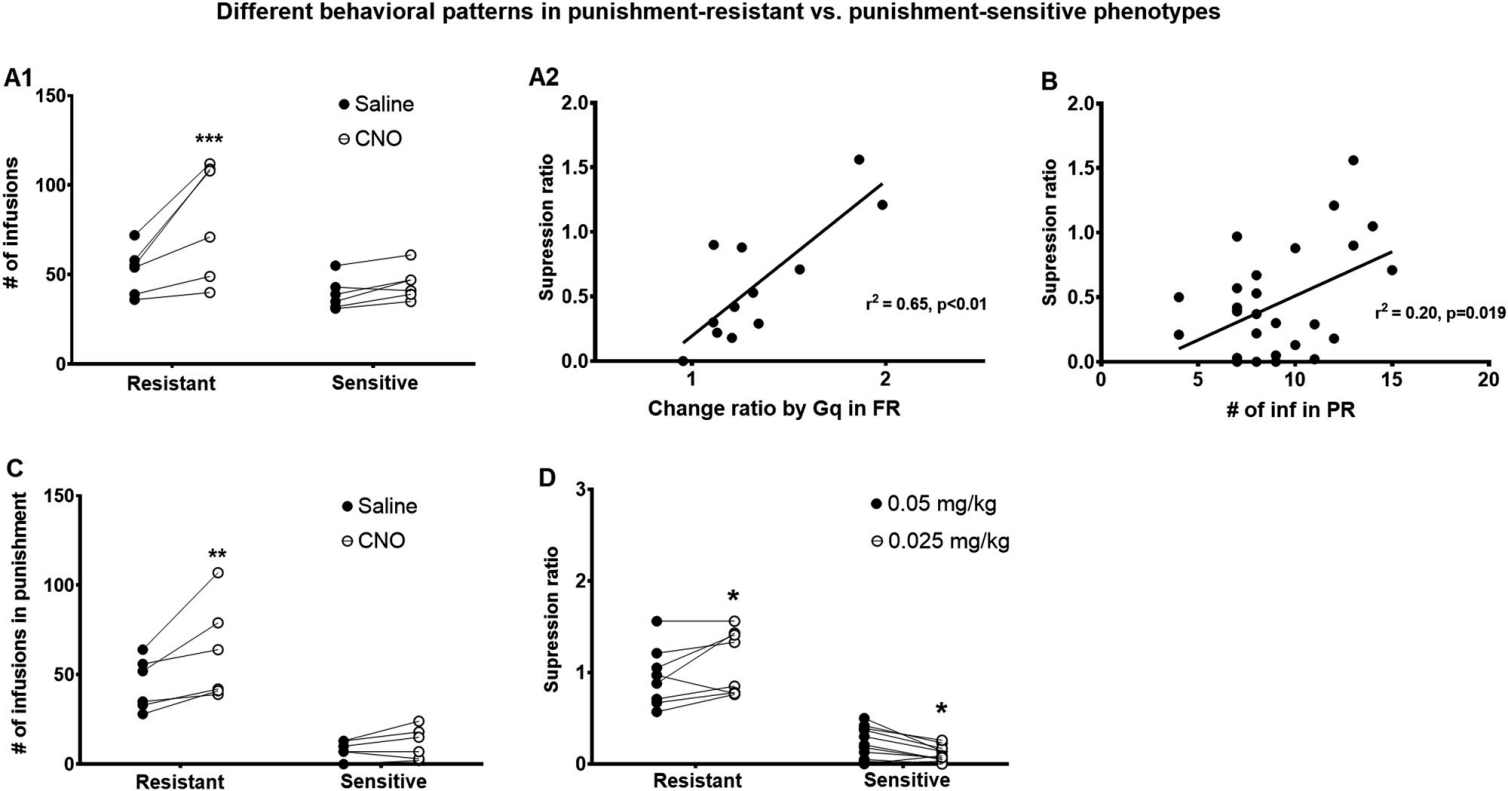
Rats with punishment-resistant and punishment-sensitive phenotypes responded differently to chemogenetic activation of DRN 5-HT neurons in FR and punishment as well as lowering the dose of heroin during punishment. **A** In FR self-administration, activating DRN 5-HT neurons increased heroin intake in punishment-resistant rats only. **A1** Number of heroin infusions during FR sessions in saline- vs. CNO-treated animals in punishment-resistant and punishment-sensitive phenotypes. Asterisks indicate a significant difference between saline vs CNO in punishment-sensitive phenotype (***p ≤ 0.001, Fisher’s PLSD). **A2** Suppression ratio on the punishment day 3 correlated with the magnitude of change caused by activation of DRN 5-HT neurons in FR. **B** Suppression ratio on the punishment day 3 correlated with a number of heroin infusions in PR. **C** Activation of the DRN 5-HT neurons increased heroin intake in punishment-resistant but not punishment-sensitive rats in punishment. Number of heroin infusions in punishment sessions in saline- vs. CNO-treated animals in punishment-resistant and punishment-sensitive phenotypes. Asterisks indicate a significant difference between saline vs CNO in punishment-resistant phenotype (** p ≤ 0.01, Fisher’s PLSD). **D** Lowering the dose of heroin produced divergent effects in punishment-resistant and punishment-sensitive rats, increasing the suppression ratio in punishment-resistant rats but decreasing the suppression ratio in punishment-sensitive rats (*p ≤ 0.05, Fisher’s PLSD).

Because of the distinct behavioral phenotype of punishment-resistant and punishment-sensitive rats, we analyzed Gq effects in punishment-resistant (*n* = 6) and punishment-sensitive (*n* = 6) rats separately in FR, PR, and punished self-administration (Fig. 4). In FR self-administration, activating DRN 5-HT neurons increased heroin intake in punishment-resistant rats only (4A1). Two-way repeated-measures ANOVA of infusion number revealed an interaction between punishment phenotype (punishment-resistant vs. punishment-sensitive) and session (saline vs. CNO) [F (1, 10) = 6.734, *p* ≤ 0.05] and main effects of punishment phenotype [F (1, 10) = 15.15, *p* ≤ 0.01] and session [F (1, 10) = 6.430, *p* ≤ 0.05]. Post hoc Fisher’s PLSD tests showed a significant difference between CNO and saline treatment in the punishment-resistant group (p ≤ 0.001) but not in punishment-sensitive group (*p* > 0.05). In the CNO session, drug intake in punishment-resistant was significantly higher than punishment-sensitive (*p* ≤ 0.01). Simple linear regression showed that the magnitude of change in heroin intake induced by chemogenetic activation (Gq) of 5-HT DRN neurons in FR correlated with suppression ratio on punishment day 3 (r^2^ = 0.65, *p* ≤ 0.01) (4A2), indicating that the response to chemogenetic activation in basal heroin self-administration predicted punishment resistance.

In PR, chemogenetic activation (Gq) of 5-HT DRN neurons had no effect in either punishment-resistant or punishment-sensitive groups (Fig S3B); however, heroin intake (motivation) correlated with suppression ratio on punishment day 3 (r^2^ = 0.20, *p* ≤ 0.05, *n* = 27) (4B), indicating that motivation for heroin predicted punishment resistance. No correlations between other experimental endpoints were found (Fig S4).

In punishment, chemogenetic activation (Gq) of 5-HT DRN neurons increased drug intake in punishment-resistant but not punishment-sensitive groups (4C). Two-way repeated-measures ANOVA of infusion number revealed an interaction between punishment phenotype (punishment-resistant vs. punishment-sensitive) and session (saline vs. CNO) [F (1, 10) = 4.87, *p* ≤ 0.05] and main effects of punishment phenotype [F (1, 10) = 24.3, *p* ≤ 0.001] and session [F (1, 10) = 10.2, *p* ≤ 0.01]. Post hoc Fisher’s PLSD tests showed a significant difference between CNO and saline treatment in the punishment-resistant group (*p* ≤ 0.01) but not in the punishment-sensitive group (*p* > 0.05). Drug intake in punishment-resistant was significantly higher than punishment-sensitive in both saline and CNO sessions (both *p* ≤ 0.001). When lowering the dose of heroin in punishment, punishment-resistant (*n* = 8) and punishment-sensitive rats (n = 15) diverged even further, with punishment-resistant rats increasing and punishment-sensitive rats decreasing their heroin intake (4D). Two-way repeated measures ANOVA of suppression ratio revealed a main effect of stress phenotype [F (1, 21) = 95.07, *p* ≤ 0.001], and an interaction between dose and stress phenotype [F (1, 21) = 12.82, *p* ≤ 0.01], but no main effect of dose [F (1, 21) = 0.9314, *p* > 0.05]. Post hoc Fisher’s PLSD tests showed that infusion numbers differed between the low and high dose of heroin in both punishment-resistant and punishment-sensitive rats (*p* ≤ 0.05).

## Discussion

Understanding the neurobiological mechanisms underlying the transition from drug use to drug addiction is critical to the development of effective therapeutic strategies for fighting the ongoing opioid crisis. In the current study, we identified a subset of punishment-resistant rats that take heroin despite negative consequences, modeling opioid use disorder in humans [36, 37]. By contrast, punishment-sensitive rats moderate their drug-taking in the face of negative consequences, similar to humans who do not progress to addiction [36, 37]. The two phenotypes did not differ in either their initial self-administration behavior or pain sensitivity. Although the intrinsic excitability of DRN 5-HT neurons was not associated with the punishment phenotype, exogenously activating DRN 5-HT neurons increased heroin intake during basal and punished self-administration only in the punishment-resistant phenotype. Moreover, rats with greater responses to chemogenetic activation of DRN 5-HT neurons in basal self-administration and rats with increased motivation for heroin in the PR test were more resistant to the footshock in punishment. Therefore, our data suggest that the DRN 5-HT system impacts heroin self-administration in a phenotype-specific (punishment-resistant) manner, linking the 5-HT system to compulsive heroin consumption.

### Punishment model for addiction-like behavior

Previous studies showed that electric shock contingent with opioid-taking or -seeking with high enough intensity is capable of suppressing opioid self-administration entirely in rats [38–40] Additionally, non-contingent (involuntary) footshock stress can reinstate extinguished heroin seeking [41, 42]. Our study, to the best of our knowledge, is the first to show that in the presence of mild contingent footshock, rats self-administering heroin can be separated into punishment-resistant or punishment-sensitive phenotypes. This finding is particularly notable in experiment 1 since 100% of lever presses were punished in this study, rather than the probabilistic schedules that are typically employed in the literature [7]. The punishment model has been used for a variety of classes of drugs, including psychostimulants, alcohol, and other opioids (oxycodone) [10–13, 15, 16, 43] as well as natural rewards [44, 45]. Similar to these previous studies, FR self-administration suggested that both phenotypes were equally sensitive to heroin reward while pain threshold data suggested no differences in sensitivity to footshock. Therefore, punishment insensitivity may rather reflect a failure of instrumental control: either a failure of punishment contingency learning or a failure to behaviorally respond to that learning [44, 45]. Interestingly, when the test dose of heroin was lowered in basal self-administration, both punishment-resistant and punishment-sensitive rats titrated their responses: increasing their number of infusions to compensate; however, when the dose was lowered in punishment, only punishment-resistant rats increased the number of infusions to compensate while punishment-sensitive rats dropped their drug intake to an even lower level. These divergent responses during punishment support the idea that punishment-sensitive rats are able to dynamically adjust drug-taking behavior by comparing reward vs. aversion outcomes while punishment-resistant rats persist in their drug-taking despite the negative consequence. Heroin intake in PR correlated with the suppression ratio in punishment, suggesting that rats with higher motivation for heroin were more prone to be punishment-resistant. This correlation is consistent with the rodent model in which prolonged exposure to addictive drugs elevates motivation and resistance to negative consequences, both hallmarks of addiction [46–49]. Whether the prolonged exposure to addictive drugs impairs instrumental control and produces compulsive, addiction-like behavior in specific populations requires further study.

### Neuronal excitability and punishment phenotype

Electrophysiological data showed that the excitability of DRN 5-HT neurons was not associated with punishment phenotypes and was not affected by heroin history. Our previous studies in a stress-hyperresponsive rat line [50] or rats exposed to stress-induced reinstatement [31, 51] show increased inhibitory GABAergic synaptic activity in DRN 5-HT neurons, and rats exposed to adolescent social isolation stress (under revision) demonstrate depressed DRN 5-HT neuronal excitability. However, these electrophysiological changes were not observed in the current study. Other electrophysiological studies have identified other brain regions that contribute to compulsive drug-taking during punishment. For example, Chen et al. found that the firing frequency of prelimbic cortex pyramidal neurons is depressed with prolonged cocaine self-administration, and rescuing the hypoactivity suppresses compulsive drug taking during punishment but not basal self-administration [11]. Decreasing central amygdala (CeA) neuronal excitability by the GABA_B_ agonist baclofen suppresses compulsive alcohol taking in punishment-resistant rats [52]. Moreover, other studies have identified differential gene expression patterns in the striatum of punishment-resistant and punishment-sensitive subjects [15, 53, 54]. Since DRN 5-HT neurons project to and receive reciprocal innervation from all of these brain regions [55, 56], and our chemogenetic data suggests the involvement of the DRN 5-HT neurons in compulsive heroin self-administration, how DRN 5-HT neurons and their afferent and efferent projections influence punishment phenotypes warrants further investigation.

### DRN 5-HT system, reward and aversion

Effects of chemogenetic activation of DRN 5-HT neurons including increased basal and punished heroin self-administration could not be explained solely by either decreased reinforcer efficacy or decreased responses to the aversive footshock during punishment. Although the link between the DRN and both drug and natural reward has been intensively investigated, the exact role of 5-HT neurons in reward is still under debate [reviewed in [18, 19, 57–60]. Optogenetic stimulation of DRN 5-HT neurons in mice produces discrepant results as some studies report reinforcing effects using self-administration and CPP models [22, 61] while others report that activation of DRN 5-HT neurons increases the waiting time for reward [24, 62]. During basal self-administration in our study, chemogenetic activation of DRN 5-HT neurons increased drug intake selectively in the punishment-resistant subgroup while lowering the dose of heroin was able to increase drug intake in all rats. Moreover, in punished conditions, punishment-sensitive rats showed a different response to chemogenetic activation than they did to a lower dose of heroin. Collectively, these data argue against decreased reinforcer efficacy as the sole explanation for chemogenetic effects in our study.

There is also literature supporting the role of the 5-HT system in aversive responding. Manipulation of DRN 5-HT neurons has been shown to alter anxious behavior and conditioned fear responses [63–65]. Consistent with a previous study in mice [21], a preliminary fiber photometry study in our lab using Tph2-iCre rats indicates that DRN 5-HT neurons are inhibited by footshock (unpublished). This inverse relationship suggests that exogenous stimulation of 5-HT neurons could have reduced the aversiveness of the footshock in our study, increasing the punished responding for heroin. However, chemogenetic activation of DRN 5-HT neurons during punishment did not change drug-taking behavior in the punishment-sensitive phenotype suggesting chemogenetic effects on footshock responses do not fully explain our findings.

### DRN 5-HT system, impulsivity, decision-making, and drug motivation

The 5-HT system and particularly its projections to the prefrontal cortex is also known to impact impulsivity and decision-making, both implicated in addiction [57]. Therefore, chemogenetic activation of the system could have increased heroin self-administration in our study via elevated impulsivity or impaired decision-making. In vivo, optogenetic manipulations of the DRN 5-HT system in mice, however, indicate that stimulation of these neurons reduces impulsivity [66] and promotes patience for future rewards [24, 67] whereas inhibition impairs decision-making [66], neither of which is consistent with the compulsive phenotype of punishment-resistant rats in response to chemogenetic activation of DRN 5-HT neurons in the current study.

Another important consideration of chemogenetic effects is that activation 5-HT DRN neurons could have increased motivation for the drug, elevating heroin self-administration under basal and punished conditions. Indeed, increased motivation for heroin in the PR task significantly predicted punishment resistance and it was the punishment-resistant phenotype that was uniquely responsive to chemogenetic manipulations of the 5-HT system. On the other hand, activation of DRN 5-HT neurons in PR did not increase breakpoint in either punishment-resistant or punishment-sensitive phenotypes as would be predicted by the motivation hypothesis. This prediction, however, depends on a breakpoint in the PR test as a reliable index of motivation for heroin, which is generally less precise for opioids compared to psychostimulants [32, 68], potentially due to sedating effects of higher opioid doses. Also, serotonergic manipulations have been reported to have discrepant effects on FR and PR responses [68]. For example, the depletion of forebrain serotonin does not affect the rate of cocaine intake but increases breakpoints in PR [69]. Given these limitations of the PR model for opioids, it remains still possible that increased motivation for heroin from activation of 5-HT DRN neurons contributed to some of the elevations of heroin self-administration seen in the current study.

### Limitations of current study and future directions

We want to acknowledge a few limitations of the current study. First, chemogenetic modulation was performed on one dose of heroin. In our future studies, different doses of heroin will be examined to thoroughly evaluate the shift in heroin dose-response curve. Second, no PR was tested in Sprague-Dawley rats in experiment 1 to verify the correlation between motivation and punishment resistance, and only males were tested in the entire study. To address those limitations, ongoing studies in our lab are comparing sex differences in Sprague-Dawley rats with FR, PR, punished self-administration as well as footshock stress-induced heroin reinstatement. Last, the DRN is a heterogenous structure and sends 5-HT projections to several brain regions involved in diverse physiological and behavioral functions [55, 56]. For example, amygdala-projecting DRN 5-HT neurons promote anxiety-like behavior, whereas frontal-cortex-projecting neurons promote active coping and anticipation of reward [21, 63, 64, 70]. Therefore, the behavioral changes in the punishment could be the net effects of activating the DRN 5-HT neurons projecting to different brain regions with opposing functions. We plan to use fiber photometry and chemogenetic tools to dissect the function of subpopulations of DRN 5-HT neurons projecting to specific brain regions in reward and aversive responses.

## Conclusions

In summary, the current study showed that rats self-administrating heroin separated into punishment-resistant and punishment-sensitive phenotypes when footshock was introduced as a contingent punishment. Although the intrinsic excitability of the DRN 5-HT neurons was not a determinant of these phenotypes, exogenous chemogenetic activation of their activity elevated both basal and punished heroin self-administration selectively in punishment-resistant rats. These data support a novel link between the DRN 5-HT system and compulsive consumption of heroin.

## Supplementary information

Supplementary information
