# Supplementary material for: Role of serotonin neurons in the dorsal raphe nucleus in heroin self-administration and punishment
Source: Neuropsychopharmacology. 2024 Sep 19;50(3):596–604. doi: 10.1038/s41386-024-01993-1 (PMC11735851; doi:10.1038/s41386-024-01993-1)
Supplement: Supplementary file 1 — Supplementary information [file 41386_2024_1993_MOESM1_ESM.pdf]

Supplementary Figures

Fig. S1 Results of TwoStep Cluster analysis. Punishment-resistant and punishment-sensitive phenotypes are well separated by using suppression ratio data from punishment day 5 in experiment 1 and from punishment day 3 in experiment 2 as inputs.

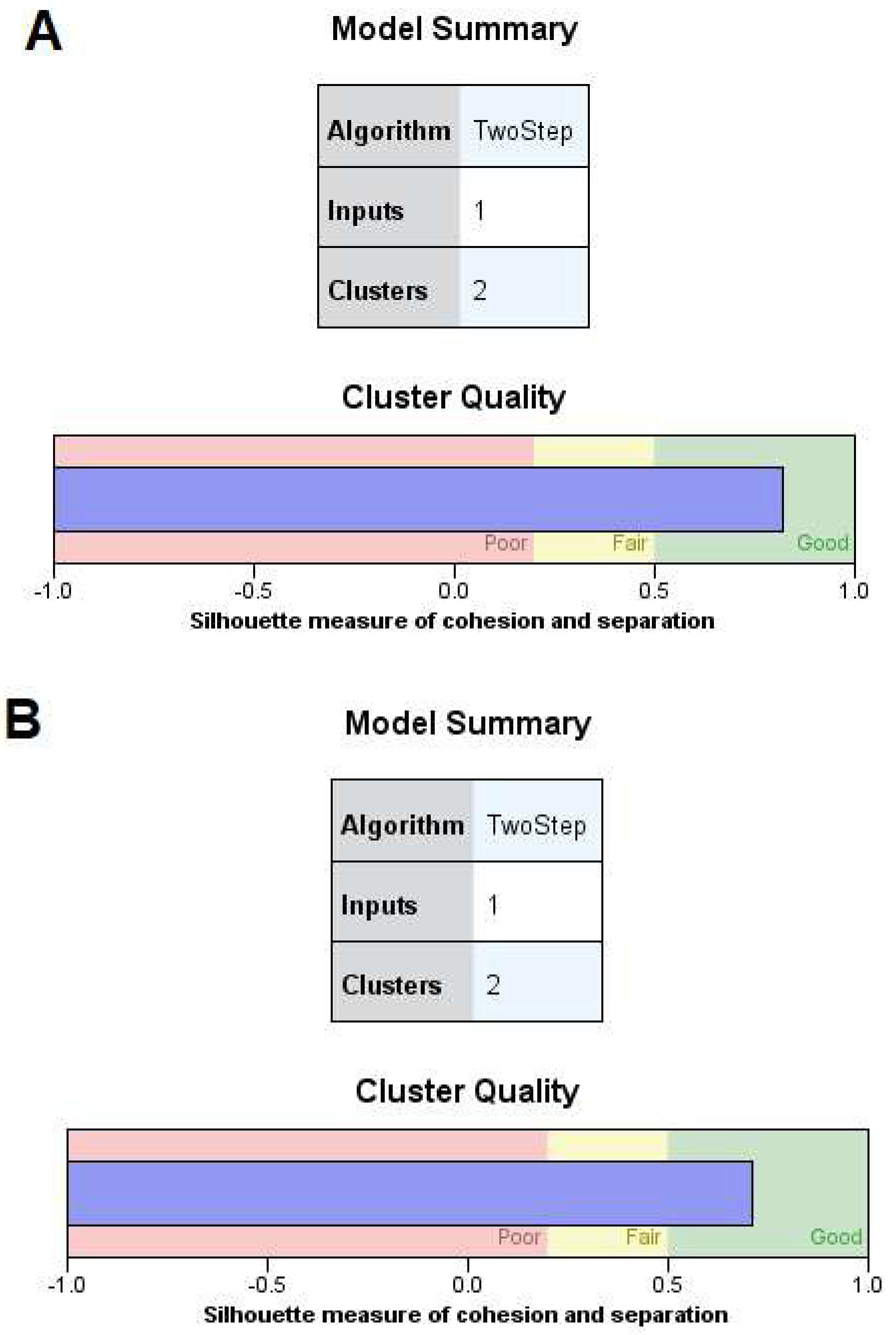

Fig. S2 Infusion numbers in punishment phase. Animals with the punishment-resistant phenotype maintained their number of infusions while those with the punishment-sensitive phenotype significantly reduced their infusions over the course of punishment. Pound signs indicate that heroin intake in punishment-sensitive rats significantly dropped from punishment day 1 (### $p \leq 0.001$). Asterisks indicate significant difference between punishment-resistant and punishment-sensitive rats on punishment days (** $p \leq 0.01$, *** $p \leq 0.001$).

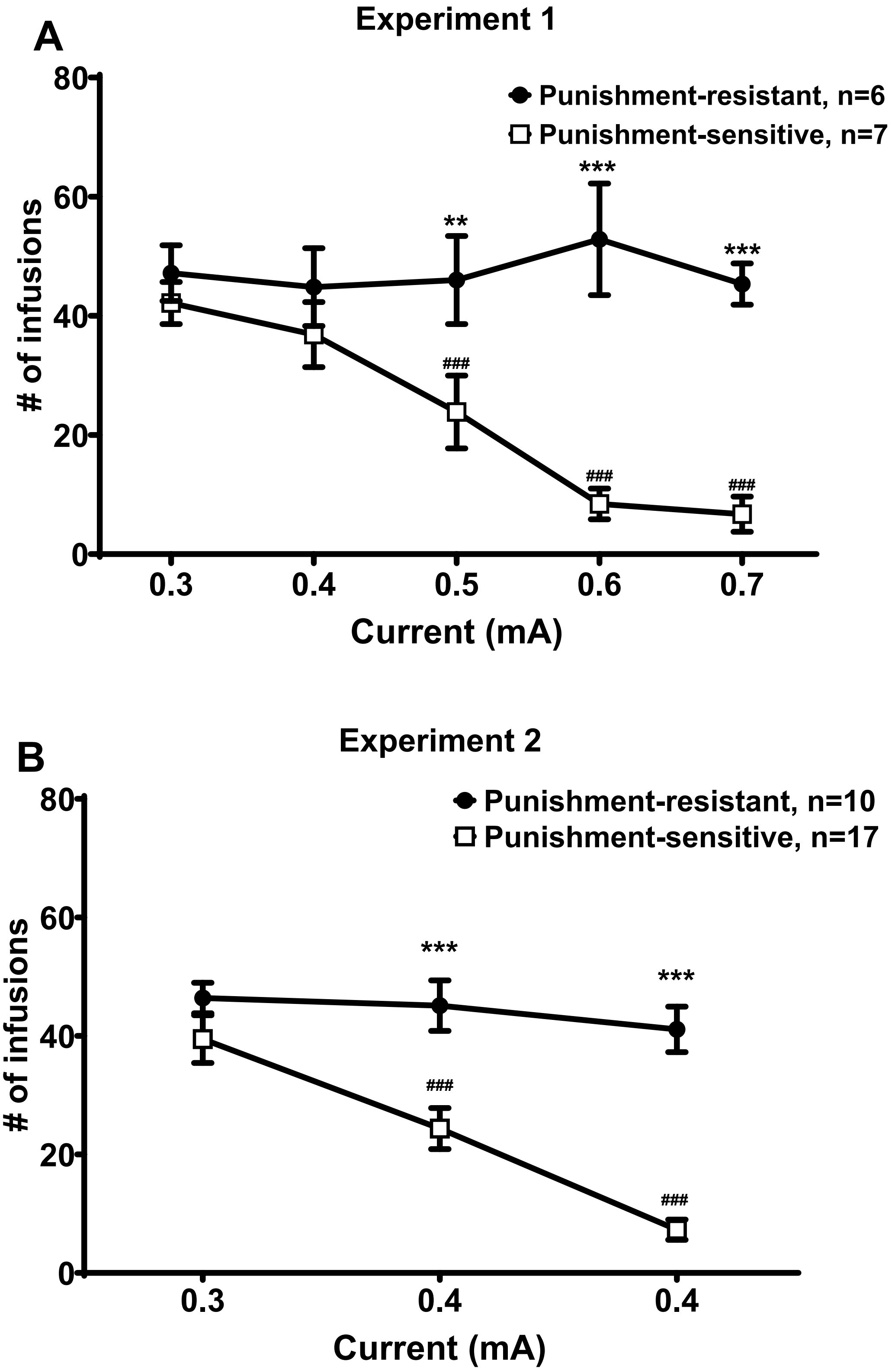

Fig S3 Breakpoint of PR. Chemogenetic manipulations did not change motivation for heroin as measured by the PR breakpoint.

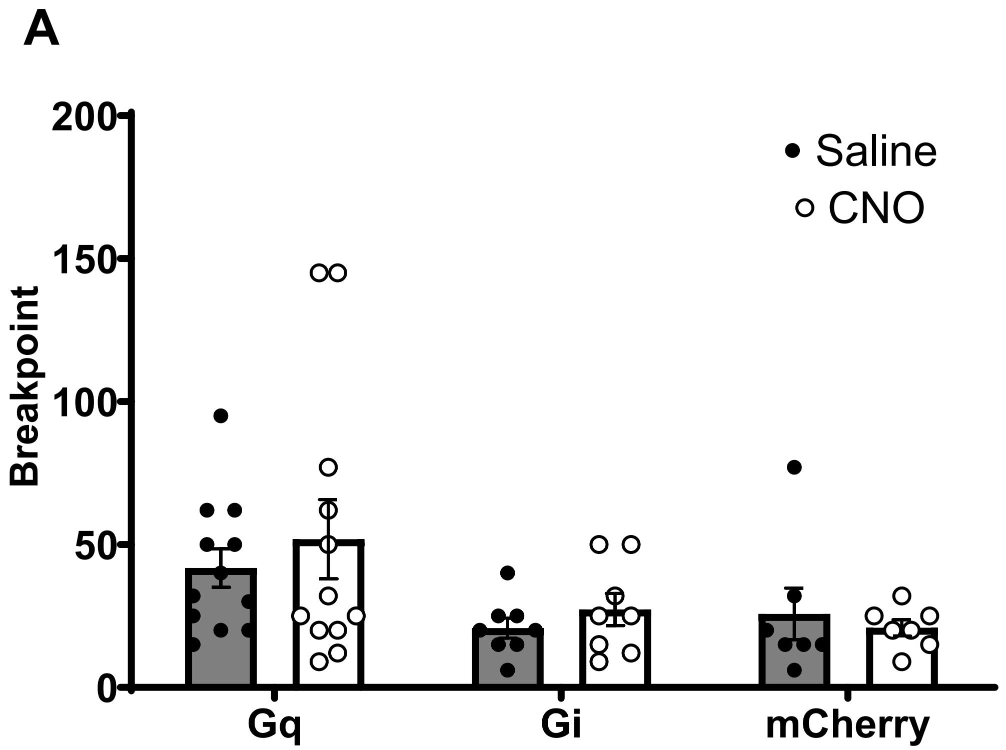

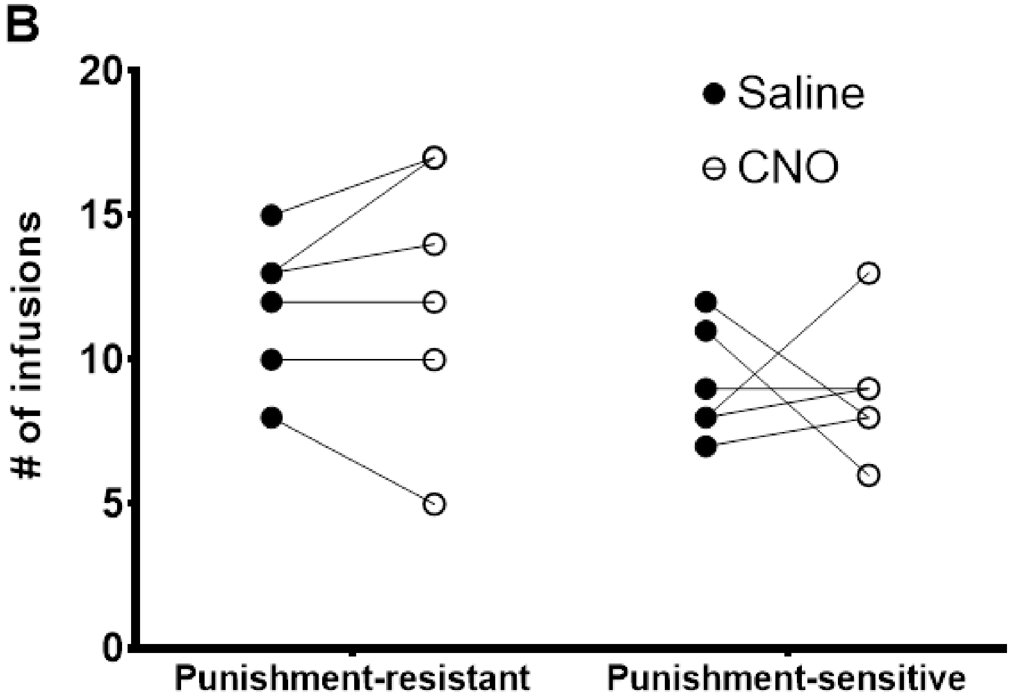

Fig S4 Resistance to punishment did not correlate with heroin intake in FR or Gq effects in PR. Effect of Gq in FR did not correlate with effect of Gq in punishment.

A

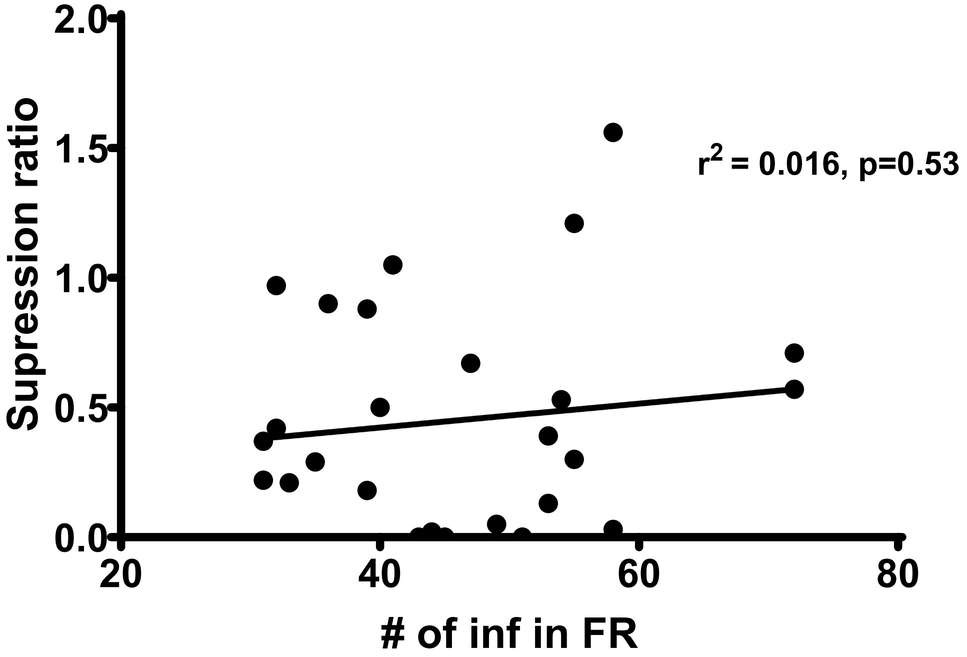

B

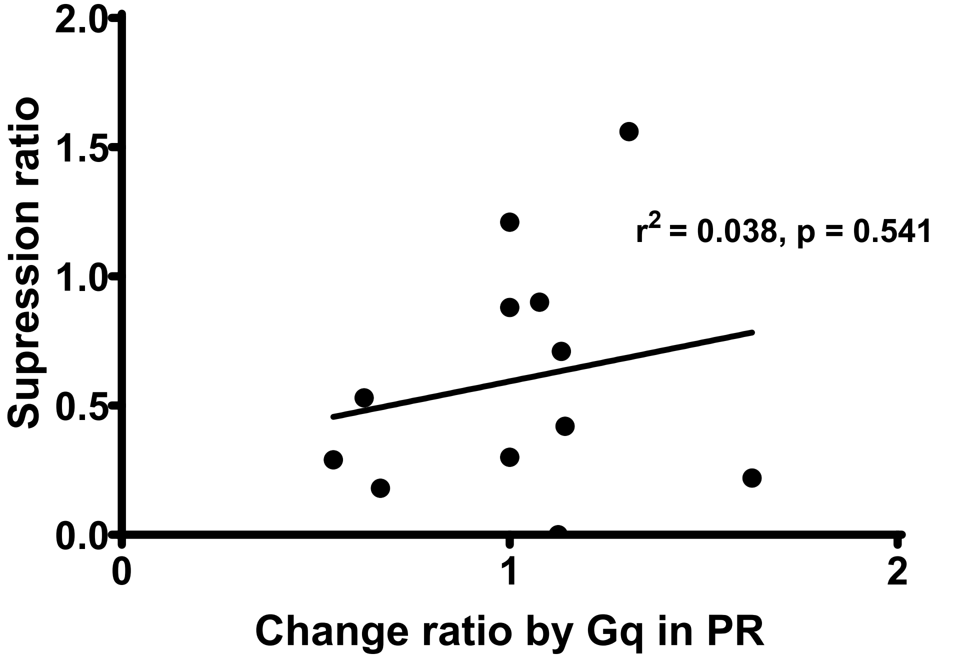

C

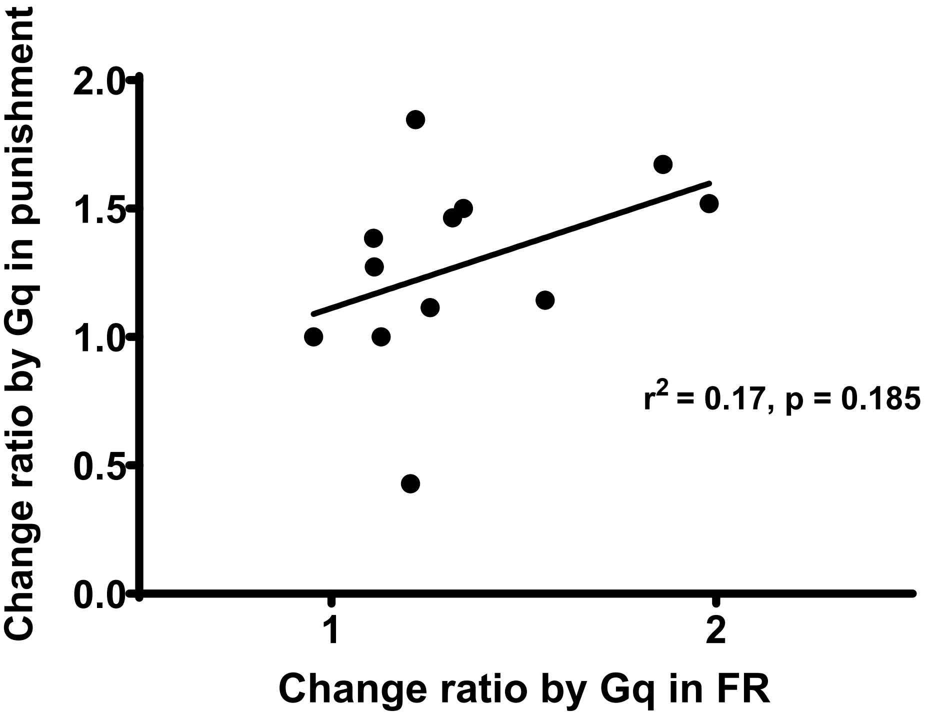

Change ratio by Gq in punishment
2.0
1.5
1.0
0.5
0.0
1
2
Change ratio by Gq in FR
r2 = 0.17, p = 0.185

Fig S5 Representative image of viral infusion showing mCherry expression confined to the DRN

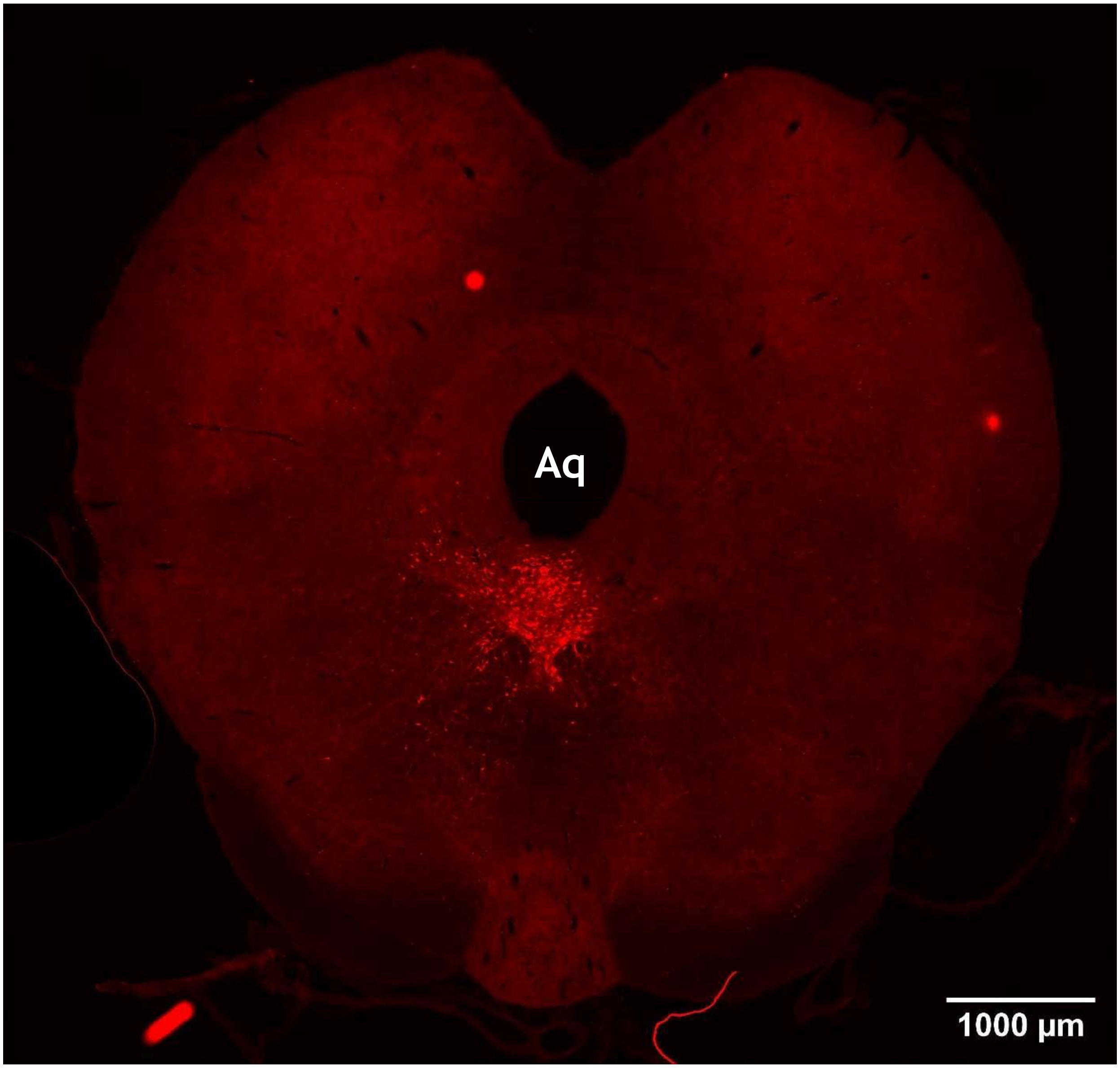

Supplemental Methods

Self-administration

Self-administration was conducted in the dark phase using operant conditioning chambers enclosed in sound-attenuating, ventilated environmental cubicles (Med Associates, Inc., St. Albans, VT). To start each session, a house light turned on and two levers extended into the chamber. Active lever presses activated the infusion pump and 2s tone-light cues, as well as footshock in punishment sessions. Drug delivery, the cue light and house light were not present during a 20 s time-out (TO20) after each infusion. Presses on the inactive lever had no consequences. Drug delivery and data collection were controlled by Med Associates software (Med PC IV). During the fixed ratio 1 (FR1) schedule, a drug infusion follows every lever press except presses during time-out periods. During the progressive ratio (PR) schedule, rats need to perform higher numbers of active lever presses to receive the next infusion of heroin. The schedule was as follows: 1, 2, 4, 6, 9, 12, 15, 20, 25, 32, 40, 50, 62, 77, 95, 118 etc. [32]. The session was aborted if the rats did not acquire any infusion within 30 min or if the 6 h maximum session duration was reached. Breakpoint was defined as the maximum number of presses resulting in the final infusion of heroin.

Measurement of paw withdrawal threshold

Paw withdrawal thresholds were measured to define pain sensitivity as previously described [33]. Rats were placed in square plexiglass boxes that contain an elevated metal mesh floor and allowed to habituate to the box for 30 minutes prior to testing. A series of von Frey filaments with logarithmically incrementing stiffness (Stoelting, Chicago, IL) was applied perpendicular to the midplantar region of both hind paws. The monofilaments were applied with increasing force until the rat withdraws the paw. Each monofilament was applied three times 10 sec apart. The

threshold was taken as the lowest force that evoked a brisk withdrawal response to one of the three repetitive stimuli. An average of two paw readings was recorded.

Electrophysiology

The day after the final behavioral session, rats were euthanized, and coronal brain slices containing the DRN were prepared for electrophysiology as described previously [31]. Slices (250 μM) were cut on a Vibratome 3000 Plus (Vibratome, Bannockburn, IL) and placed in ACSF (35°C) ((in mM): 124 NaCl, 2.5 KCl, 2 NaH_2PO_4, 2.5 $CaCl_2$, 2 $MgSO_4$, 10 dextrose, and 26 $NaHCO_3$) with l-tryptophan (50 mM)) bubbled with 95% O_2/5%CO_2 for 1h. Slices were transferred to a recording chamber (Warner Instruments, Hamden, CT) and perfused with oxygenated ACSF at 1.5-2.0 ml/min. DRN neurons were visualized using a Nikon E600 upright microscope (Optical Apparatus, Ardmore, PA). Whole-cell patch-clamp recordings were conducted with a HEKA patch-clamp EPC-10 USB amplifier (HEKA Elecktronik Lambrecht, Pfalz, Germany) in current-clamp mode (I=0 pA). The resistance of the electrode was 4–6 MΩ filled with intracellular solution (in mM):120 Kgluconate, 10 KCl, 2 $MgCl_2$, 10 EGTA, 10 HEPES, 2 MgATP, 10 NaPhosphocreatine, 0.5 Na_2GTP, 0.1% biocytin, pH 7.3. Series resistance was monitored throughout the experiment and the cell discarded if the series resistance was unstable or exceeded four times the electrode resistance. Signals were filtered at 1 kHz and digitized at 10 kHz. The liquid junction potential was −9 mV between the pipette solution and the ACSF and was not subtracted from the data obtained. Only one cell per slice was recorded in the ventromedial subdivision of the DRN at mid-caudal levels that correspond to 7.32–8.16 mm caudal to bregma where 5-HT neurons are most densely populated. Electrophysiology data were analyzed with Patchmaster software (HEKA).

Immunohistochemistry

Following electrophysiology experiments, brain slices were post fixed in 4.0% paraformaldehyde for 20–24 h and kept in 30% sucrose solution until standard dual fluorescence immunohistochemical methods were performed to visualize the recorded biocytin-filled cell and Tph2 immunoreactivity as a marker of 5-HT neurons [31]. A rabbit-anti Tph2 antibody (1:500; Millipore, Billerica, MA) was visualized using an Alexa 594-conjugated donkey anti-rabbit secondary antiserum (1:1000; Life Technologies, Carlsbad, CA). Biocytin was visualized using Alexa 488-conjugated streptavidin (1:1000; Life Technologies, Carlsbad, CA). For DREADD function studies, the expression of mCherry was confirmed for biocytin-filled cells.

To verify the 5-HT neuron-specific expression of Cre recombinase, tamoxifen-induced Tph2-iCre rats were anesthetized and perfused with phosphate-buffered saline followed by 4% paraformaldehyde. Brains were removed, post-fixed for 24 h, cryoprotected with 30% sucrose until sectioning. Slices containing the DRN were sectioned by cryostat at 50 μm thickness and stained with mouse anti-Cre Recombinase (1:1000; Millipore, Billerica, MA) and donkey anti-mouse Alexa Fluor 488 (1:1000; Millipore, Billerica, MA) as well as with rabbit anti-Tph2 (1:500) primary antibody and donkey anti-rabbit Alexa Fluor 594 (1:1000) secondary antibody.

The slices were coverslipped with SlowFade Gold mounting medium (Life Technologies, CA) and visualized with Nikon A1R Confocal Scanning System with NIS Elements AR imaging software. The DRN slices from the Tph2-iCre rats in experiment 2 were visualized after behavioral tests to confirm the expression of DREADDs and mCherry. Animals with weak or off-target mCherry expression were excluded from analysis.
